# Periodontal Disease and Pregnancy Outcomes

**DOI:** 10.1155/2010/293439

**Published:** 2010-08-12

**Authors:** Dolapo A. Babalola, Folashade Omole

**Affiliations:** Department of Family Medicine, Morehouse School of Medicine, 1513 E Cleveland Avenue Bldg 100, Ste 300, East Point, GA 30344, USA

## Abstract

An increasing number of studies are confirming an association between periodontal disease (PD) and adverse outcomes in pregnancy. PD places pregnant women at greater risk for preterm birth than alcohol consumption or smoking. This underscores the importance of offering dental screening to women who are pregnant or contemplating pregnancy and the need for physicians who provide obstetric care to be aware of the possible connection between poor dental health and poor pregnancy outcomes.

## 1. Introduction

An increasing number of studies are confirming an association between periodontal disease (PD) and adverse outcomes in pregnancy. Offenbacher et al. found that pregnant women with severe PD are 7.5 times more likely to go into labor prematurely. PD places pregnant women at greater risk for preterm birth than alcohol consumption or smoking [1]. We report on a 29-year-old pregnant patient with PD who experienced a spontaneous abortion at 19-week gestation. We hypothesize that this case may mirror the effect seen between periodontal disease and adverse pregnancy outcome such as spontaneous preterm birth. Though studies refer to premature birth, we postulate that this might have been our patient's scenario if the pregnancy was advanced in gestational age. This underscores the importance of offering dental screening to women who are pregnant or contemplating pregnancy and the need for physicians who provide obstetric care to be aware of the possible connection between poor dental health and poor pregnancy outcomes.

## 2. Case

**Figure 1:**
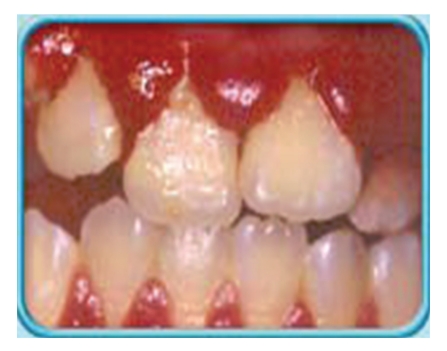
Pregnancy Gingivitis.

**Figure 2:**
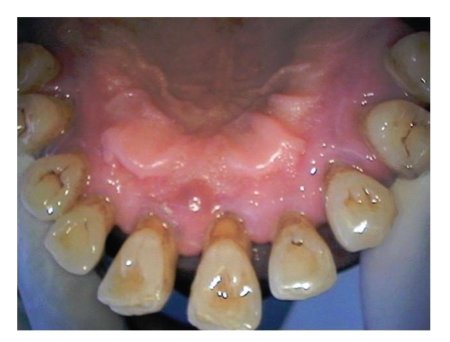
Periodontal disease.

A 29-year-old gravida 6, para 2032 presented at 19-week gestation with fluid leakage. She denied history of trauma, smoking, alcohol, or illicit drug use. Her medical history was significant for chronic gingivitis (Figure 1) which progressed to periodontal disease (Figure 2). Prior to pregnancy, patient had follow-up appointments with her dentist for which she was diagnosed with mild to moderate periodonitis through a comprehensive examination. This included an evaluation of soft tissue, bleeding and exudate on probing. She underwent treatment which involved surgical debridement of the necrotic tissue. During her prenatal visit two weeks prior to presentation, she was treated with antibiotics when she complained of painless gum redness and swelling and easy bleed with contact. 

Otherwise her current and previous pregnancies were uneventful including negative triple screen. On admission, her vitals were stable, and fetal Doppler heart rate was between 140 and 150. Her physical exam was positive for multiple caries in her right lower molars, the gingival margin was red and swollen and easy bleeding occurred with light contact. The patient's abdomen was nontender, with the fundal height at the umbilicus. Pelvic exam revealed fluid leakage from dilated cervix. A sonogram of the fetus done later demonstrated no cardiac activity and severe oligohydramnios. The patient was diagnosed with inevitable abortion and delivered a stillborn, female fetus less than 7 hours after vaginal insertion of dinoprostone. Genetic screening of the fetus was negative for chromosomal abnormalities

## 3. Discussion

Preterm birth (PTB) complicates 12% of all pregnancies in the US which is one of leading causes of infant morbidity and mortality. Maternal infection such as periodontal disease can play a role in PTB, though this is still a controversial topic. Periodontal disease is divided into two categories; Gingivitis is a mild, reversible inflammation of the gingival tissues and Periodontal disease is a more severe and destructive irreversible form of the disease. 80% of American adults are affected with some form of periodontal disease [1].

Although recent studies have concluded that the etiology of 25% to 50% of preterm low birth weight (PLBW) deliveries is unknown, growing evidence indicates that diverse degrees of periodontal infection may play a significant role [1]. 

Offenbacher et al. conducted a cross-sectional study that showed women who gave birth to PLBW babies had significantly higher levels of periodontal pathogens in their subgingival plaque, compared with women whose babies were normal weight [1]. In their cross-sectional study, Offenbacher et al. measured the levels of PGE_2_ and IL-1 beta in the gingival crevicular fluid (GCF) of pregnant women [1]. 

GCF originates from the epithelium of the gingival crevice and helps fight infection by ferrying immunoglobulins, antibodies, and other substances between the connective tissue and the subgingival space; the GCF flow rate increases in response to inflammation of gingival tissue. Offenbacher et al. determined that the amounts of PGE_2_ and IL-1 beta in the GCF related inversely to birth weight; thus, women with higher levels of PGE_2_ and IL-1 beta in their GCF delivered smaller babies, overall, and were more likely to give birth prematurely [1]. 

 In a study of 1313 pregnant women, Jeffcoat et al. found that the risk of preterm delivery was 4 to 7 times greater for women with generalized perionditis [2]. Oral bacteria associated with PD, such as Bacteroides forsythus, Fusobacterium nucleatum, and Porphyromonas gingivalis have been implicated in preterm birth [2]. Jeffcoat et al. conducted an interventional study that provided nonsurgical periodontal therapy to a group of women who were between 21 and 24 weeks gestation [2]. Results were compared with a control group of pregnant women, who received no therapy. The cohort that received dental treatment had a preterm birth rate of 0.8% versus 6% for the untreated group. This supports the theory that periodontal infections may play a role in many instances of PLBW and that PD is a major risk factor for preterm delivery [2].

Although the many advances in medicine, the rate of preterm birth has not decreased in the United States over the past several decades. In fact, the rate rose in 2003 to more than 12% of all births in the United States. This equates to over half a million premature births in the United States alone [3]. Consequently, the identification of risk factors for preterm birth which are amenable to intervention would have far-reaching and long-lasting effects. Jeffcoat et al. conducted a systematic review of 31 studies, and 22 of these identified a correlation between PD and adverse pregnancy outcomes such as premature birth [3]. 

Addressing PD during pregnancy may decrease a woman's risk of preterm birth. López et al. found that the incidence of preterm birth in women whose teeth were treated with scaling or root planting during pregnancy was <2%. Women who postponed dental treatment until after delivery had a preterm birth incidence of 10% [4].

**Figure 3:**
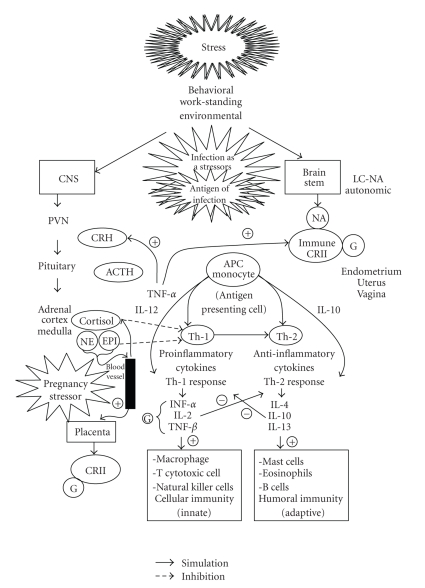
Environmental stressors and pregnancy.

One theory proposes that periodontal infection increases the risk of premature labor by accelerating prostaglandin E_2_ (PGE_2_) production. In a normal pregnancy, PGE_2_ production in the amnion increases gradually throughout the gestational period. Labor is triggered once the level of PGE_2_ reaches a certain threshold [5]. Analyses of amniotic fluid in pregnant women with PD have identified various bacterial products, such as lipopolysaccharide and enzymes from gram-negative bacteria, that are known to stimulate the production of proinflammatory cytokines. This results in higher levels of tumor necrosis factor, interleukin (IL)-1 beta, IL-6, and PGE_2_, thereby increases the risk of a PLBW delivery (Figure 3) [5].

Studies involving the introduction of periodontopathic lipopolysaccharides into the amniotic fluid of pregnant sheep and Fusobacterium nucleatum into the amniotic fluid of mice have produced similar findings [6, 7].

A few randomized controlled trials have shown no link between treatment of periodontitis and pregnancy outcomes, although these conclusions may change following the results of large randomized controlled trials. Treatment of localized periodontal disease in pregnancy does not reduce the occurrence of preterm birth as resulted from the multicenter, randomized clinical trial. Subjects with PD were randomized to scaling and root planing (active) or tooth polishing (control). The primary outcome was the occurrence of Spontaneous preterm delivery (SPTD) at <35 weeks of gestation [8].

## 4. Conclusion

The factors involved in many cases of adverse pregnancy outcomes related to PD are still ongoing and controversial. Our case demonstrates the likely possibility that the patient's chronic history of gingivitis and subsequently developing mild to moderate periodontitis might have been related to her abortion at 19 weeks gestation. This was in the absence of the confounding variables such as smoking, drinking, or a previous history of preterm birth, and the fetal products were negative for chromosomal abnormalities. 

It is vitally important for women of reproductive age including pregnant women to practice good dental hygiene which involves making regular dentist visits that include the removal of calculus or tartar, brushing and flossing regularly, and using mouthwash. This is because pregnancy causes hormonal changes that increase the risk of developing gum disease, and because your oral health can affect the health of the developing baby. In an effort to sustain a normal pregnancy, it is necessary to balance the mother's nutritional, hormonal, and immunological systems [9].

Even though much of the literature on PD and pregnancy identifies a positive association between PD and PLBW, others are controversial perhaps fairly due to differences in study design and defining both thresholds of periodontal disease and adverse outcome. It is still vital that clinicians consider periodontal care and screening for PD an integral component of prenatal care. If PD is diagnosed, prompt and appropriate management is essential [9].
